# SmGRAS1 and SmGRAS2 Regulate the Biosynthesis of Tanshinones and Phenolic Acids in *Salvia miltiorrhiza*

**DOI:** 10.3389/fpls.2019.01367

**Published:** 2019-10-30

**Authors:** Wenrui Li, Zhenqing Bai, Tianlin Pei, Dongfeng Yang, Renjun Mao, Bingxue Zhang, Chuangfeng Liu, Zongsuo Liang

**Affiliations:** ^1^Institute of Soil and Water Conservation, Chinese Academy of Sciences and Ministry of Water Resources, Yangling, China; ^2^University of the Chinese Academy of Sciences, Beijing, China; ^3^College of Life Sciences, Northwest A&F University, Yangling, China; ^4^College of Life Sciences and Medicine, Zhejiang Sci-Tech University, Hangzhou, China

**Keywords:** *Salvia miltiorrhiza*, SmGRAS1/2, GA, tanshinines, phenolic acids, biosynthesis

## Abstract

*Salvia miltiorrhiza* is one of the most widely used traditional Chinese medicinal plants because of its excellent performance in treating heart diseases. Tanshinones and phenolic acids are two important classes of effective metabolites, and their biosynthesis has attracted widespread interest. Here, we functionally characterized SmGRAS1 and SmGRAS2, two GRAS family transcription factors from *S. miltiorrhiza*. *SmGRAS1/2* were highly expressed in the root periderm, where tanshinones mainly accumulated in *S. miltiorrhiza*. Overexpression of *SmGRAS1/2* upregulated tanshinones accumulation and downregulated GA, phenolic acids contents, and root biomass. However, antisense expression of *SmGRAS1/2* reduced the tanshinones accumulation and increased the GA, phenolic acids contents, and root biomass. The expression patterns of biosynthesis genes were consistent with the changes in compounds accumulation. GA treatment increased tanshinones, phenolic acids, and GA contents in the overexpression lines, and restored the root growth inhibited by overexpressing *SmGRAS1/2*. Subsequently, yeast one-hybrid, dual-luciferase, and electrophoretic mobility shift assays (EMSA) showed SmGRAS1 promoted tanshinones biosynthesis by directly binding to the GARE motif in the *SmKSL1* promoter and activating its expression. Yeast two-hybrid assays showed SmGRAS1 interacted physically with SmGRAS2. Taken together, the results revealed that SmGRAS1/2 acted as repressors in root growth and phenolic acids biosynthesis but as positive regulators in tanshinones biosynthesis. Overall, our findings revealed the potential value of SmGRAS1/2 in genetically engineering changes in secondary metabolism.

## Introduction

Danshen, the dried roots of *Salvia miltiorrhiza* Bunge, is a traditional Chinese medicine in treatment of cardiovascular and cerebrovascular diseases (Dong et al., 2011). In addition, it also has many pharmaceutical activities, including anti-inflammatory, antibacterial, and antiancer properties (Jiang et al., 2013). In China, numerous pharmaceutical dosage forms of Danshen are commercially available, including tablets, capsules, oral liquids, injectables, granules, and dripping pills. As a model medicinal plant with great economic and medicinal value, there has been many extensive interests in improving bioactive ingredients (Xu et al., 2015a; Xu et al., 2015b). The bioactive ingredients of *S. miltiorrhiza* fall into two main groups: hydrophilic components (phenolic acids), such as salvianolic acid B and rosmarinic acid (RA), and lipophilic components (tanshinones), such as dihydrotanshinone I (DT-I), cryptotanshinone (CT), tanshinone I (T-I), and tanshinone IIA (T-IIA) (Huang et al., 2019). The contents of tanshinones and phenolic acids are the major quality markers of *S. miltiorrhiza* medicinal materials, according to the Chinese Pharmacopoeia (The State Pharmacopoeia Commission of China, 2015). As one kind of diterpenoids, tanshinones are synthesized through mevalonic acid and 2-C-methyl-D-erythritol-4-phosphate pathways (Kai et al., 2011; Ma et al., 2015), which included *AACT*, *HMGR*, *DXS*, *DXR*, *CMK*, *GGPPS*, *KSL*, and *CYP76AH* biosynthetic genes (Ma et al., 2012). Phenolic acids are produced in phenylpropanoid and tyrosine-derived pathways (Pei et al., 2018), which included *C4H*, *4CL*, *TAT*, and *CYP98A14* biosynthetic genes (Xu et al., 2016). Many reports have focused on these key synthase genes, which could improve the accumulation of active components. However, relatively less is known about the regulatory mechanisms of transcriptional factors in the biosynthesis of tanshinones and phenolic acids in *S. miltiorrhiza*.

GA is an important phytohormone that controls many aspects of plant growth and development through GA signaling pathway (Sun, 2011). It also has been reported to regulate root growth and secondary metabolism (Du et al., 2015; Davière and Achard, 2016). GA could promote root growth of *Arabidopsis via* directly reducing the level of flavonols (Tan et al., 2019). Moreover, there is an interaction between energy metabolism and the GA-mediated control of growth that coordinates cell wall extension, lipid metabolism, and secondary metabolism in *Arabidopsis* (Ribeiro et al., 2012). The metabolic pathways of GA biosynthesis and degradation, as well as GA signaling pathways, have been reported (Sun, 2011; Du et al., 2015). As a group of diterpenoids, GA shares the universal precursor geranylgeranyl diphosphate (GGPP) with other diterpenoids, such as tanshinones (Ma et al., 2012; Du et al., 2015). The biosynthesis of tanshinones from GGPP involves *CPS1/2*, *KSL1*, *CYP76AH1/3*, and other unknown genes, while the biosynthesis of GA from GGPP involves *CPS5*, *KS*, *KAO*, *GA20ox*, *GA3ox,* and *GA2ox* genes (Ma et al., 2012; Cui et al., 2015; Su et al., 2016). Notably, GA treatment could increase tanshinones accumulation in the wild-type hairy roots of *S. miltiorrhiza* (Liang et al., 2013; Bai et al., 2017). Thereby, there may exists a tradeoff between GA and tanshinones biosynthesis in *S. miltiorrhiza*.

GRAS family transcription factors (TFs), the key regulators of GA signaling, integrated multiple signaling pathways (Hakoshima, 2018). Members of GRAS gene family have been identified in many plants, including *Arabidopsis*, rice, tomato, and grapevine (Tian et al., 2004; Huang et al., 2015; Grimplet et al., 2016). Based on amino acid sequences, the GRAS family was divided into 13 distinct subfamilies: DELLA, SCR, SHR, PAT1, SCL3, SCL4/7, LISCL, SCL28, LAS, HAM, DLT, OS4, and OS19 (Huang et al., 2015). Previous studies have reported that GRAS proteins play diverse roles in root development, GA signal transduction, light signaling, and biotic and abiotic stress responses (Livne et al., 2015; Xu et al., 2015a; Xu et al., 2015b; Heck et al., 2016). For instance, SCR and SHR formed a complex in order to participate in regulating root-related developmental processes in *Arabidopsis* (Cui et al., 2007; Lucas et al., 2011). The PAT1 subfamily had been shown to mediate phytochrome and defence signaling pathways (Hakoshima, 2018). SCL3 functioned as a repressor of DELLA, which could positively regulate the GA signaling pathway and control GA homeostasis in *Arabidopsis* root development (Zhang et al., 2011). Therefore, we speculated that SmGRAS could regulate the root development through controlling the GA homeostasis in *S. miltiorrhiza*.

Since tanshinones are mainly concentrated in the periderm of *S. miltiorrhiza* roots and induced by GA treatment (Xu et al., 2015a; Xu et al., 2015b; Bai et al., 2017), we speculated that the GA response factors SmGRASs might participate in the tanshinones biosynthesis in *S. miltiorrhiza* roots. Although five GRAS family genes have been identified in *S. miltiorrhiza* (Bai et al., 2017), how do the SmGRASs participate in root growth and diterpenoid metabolic flux remains unknown. In this study, we characterized and analyzed the functions of two GRAS genes, *SmGRAS1* and *SmGRAS2* in *S. miltiorrhiza*. Overexpression (OE) of *SmGRAS1/2* could inhibit root growth, increase the accumulation of tanshinones, and reduce the contents of GA and phenolic acids. However, all the patterns of the contents mentioned above had the opposite changes after GA treatment in the OE lines, except the tanshinones. Subsequently, yeast one-hybrid (Y1H), dual-luciferase (Dual-LUC), and electrophoretic mobility shift assay (EMSA) confirmed that SmGRAS1 could directly bind to the GARE motif in the promoter of *SmKSL1* to induce its expression. Yeast two-hybrid (Y2H) further illustrated SmGRAS1 interacted with SmGRAS2. Finally, the molecular mechanisms of the regulation of GA-mediated root growth and secondary metabolite biosynthesis by SmGRAS1/2 were analyzed and discussed. Functional analysis of SmGRAS1/2 on regulating the root growth and diterpenoid metabolic flux increases our understanding of the molecular basis of the tradeoff between GA and tanshinones biosynthesis, providing a framework for metabolic engineering in *S. miltiorrhiza*.

## Materials and Methods

### Plant Materials, Growth Conditions, and GA Treatment

The *S. miltiorrhiza* hairy roots were derived from sterile plantlets infected with *Agrobacterium rhizogenes* bacterium (*ATCC15834*), as previously reported (Ru et al., 2016). The hairy roots (0.3 g fresh weight) were cultured in 50 ml of liquid 6,7-V medium on an orbital shaker and sub-cultured every 30 days. *Nicotiana benthamiana* was grown in a greenhouse (16 h: 8 h, light: dark) at 25°C for 30 days and used for the subcellular localization experiments.

A GA_3_ (Sigma, USA) stock solution was added to the 21-day-old hairy roots to obtain a final concentration of 100 μM. The hairy roots were treated for 2 h, 24 h, or 6 days. Hairy roots without GA_3_ treatment were used as controls. The controls and treated roots were collected at the same time and used for real-time quantitative PCR (qRT-PCR) analysis and high-performance liquid chromatography (HPLC) analysis. All treatments were performed in three independent biological replicates.

To analyze the tissue-specific *SmGRAS1/2* genes expression levels, leaf, stem, flower, bud flower, phloem, xylem, and periderm tissue were collected from the 2-year-old *S. miltiorrhiza*.

### Bioinformatics Analysis of SmGRAS1/2

SmGRAS1 and SmGRAS2 protein sequences from *S. miltiorrhiza* and multiple sequence alignments of GRAS protein sequences from *Arabidopsis thaliana* (http://www.arabidopsis.org) were performed using the ClustalX program. A phylogenetic tree based on the alignment was constructed with MEGA6 by the neighbor-joining method with the bootstrap test (n = 500 replications).

### RNA Extraction and qRT-PCR Assays

Total RNA was extracted by using the RNAprep pure plant kit (TIANGEN, China), and then reverse transcribed to cDNA using the PrimeScript™ RT reagent kit (TaKaRa, China). qRT-PCR was performed on a real-time PCR system (Bio-Rad CFX96, USA) using the SYBR Premix Ex Taq II Kit (Takara, China). The *SmActin* gene was used as the endogenous control (Yang et al., 2010). The relative expression levels of the genes were calculated by the 2^−ΔΔct^ method. All the primers used for the qRT-PCR analysis are listed in **Table S1**. The data were obtained from three independent biological replicates and three technical replicates.

### HPLC Analysis of Tanshinones and Phenolic Acids Contents

The contents of tanshinones and phenolic acids in the *S. miltiorrhiza* roots were determined by HPLC, according to previous method (Liu et al., 2016). In brief, 0.04 g powder of dried hairy roots was extracted by soaking the sample overnight in 8 ml of 70% methanol and then sonicating the sample for 45 min. The mixture was centrifuged at 8000 g for 10 min, and the supernatant was filtered through a 0.2-μm filter and analyzed by HPLC.

### Subcellular Localization

The full-length coding regions of *SmGRAS1/2* were fused with green fluorescent protein (GFP) in the *pA7-GFP* vector. The *pA7*-*SmGRAS1/2-GFP* and *pA7-GFP* plasmids were transiently transformed into onion epidermis with gene gun (Bio-Rad, USA). After 1 day of incubation, the onion epidermis was stained with 4,6-diamidino-2-phenylindole dihydrochloride (DAPI) (Solarbio, China) for 20 min, washed twice with PBS buffer (pH 7.2), and later observed under a confocal laser scanning microscope (Nikon A1R, Japan).

The *pA7*0390-*SmGRAS1/2-GFP* and *pA7*0390*-GFP* plasmids were transformed into *Agrobacterium* strain *GV3101*. The *GV3101* suspension cultures were infiltrated into leaves of 4-week-old *N. benthamiana*, following the previously described method (Bai et al., 2018). After 2 days of co-culture, the protoplasts were prepared as previously described (Li, 2011). The protoplasts were stained with DAPI for 15 min and later observed under a confocal laser scanning microscope. The primers used for the subcellular localization analysis are listed in **Table S1**.

### Analysis of Transcriptional Activity

The *pDEST-GBKT7-SmGRAS1/2* and *pDEST-GBKT7* plasmids were transformed into the yeast strain AH109. The *pGBKT7-53 + pGADT7-T* plasmid was constructed as a positive control. The transformed AH109 were first screened on synthetic dropout (SD) medium lacking tryptophan (SD/-Trp) and then selected on SD medium without tryptophan, histidine and adenine (SD/-Trp/-His/-Ade). Transcriptional activity was evaluated according to the growth status of the yeast.

### Plasmid Construction and Genetic Transformation

The full-length sequences of *SmGRAS1/2* were amplified and cloned into the restriction sites *Noc*I and *Spe*I of the *pCAMBIA1304* binary vector in sense and antisense orientations under the control of the CaMV35S promoter. The positive clones were confirmed by PCR and restriction enzyme digestion. Afterwards, the plasmids were transformed into ATCC15834. The transformants were screened with a combination of cefotaxime (Sigma, USA) and hygromycin B (MP Bio, USA). Genomic DNA was isolated from hairy roots by using the cetyl trimethylammonium bromide method. Four primer pairs, *rolB*, *rolC*, *hptII*, 35S forward primer (35S F), and *GRAS1/2* reverse primer (GRAS1/2 R), were designed for the PCR identification and the positive transgenic lines screening. The positive transgenic lines were used for the qRT-PCR and HPLC analyses. All the primers used for the expression vector construction and the PCR identification of transgenic lines are listed in **Table S1**.

### Determination of GA_3_ Concentrations

The 21-day-old transgenic hairy roots and control roots (ATCC) were treated with 100 μM GA_3_ for 6 days. Each line (ATCC, ATCC-GA, G1O7, G1O7-GA, G2O17, and G2O17-GA) was collected in three biological replicates and used for the analysis GA_3_ concentrations, which were measured by HPLC as previously described (Mornya and Cheng, 2018).

### Y1H Verification

The coding sequences of full-length *SmGRAS1/2* were inserted into the *pGADT7* vector. The primers for the *pGADT7-SmGRAS1/2* vector are listed in **Table S1**. Then, the 649-bp *SmKSL1* promoter sequences were cloned into the *pBait-AbAi* vector. The primers for the bait vectors (*pBait-AbAi-SmKSL1-649*) are listed in **Table S1**. Aureobasidin A (AbA) suppressed the basal expression of the *Y1H-pAbAi-SmKSL1-649* (*PYK649*) yeast strain (Bai et al., 2018). *pGADT7-SmGRAS1/2* was verified by interactions with *PYK649* yeast strains, which recombined the *SmKSL1* promoter in SD/-Leu/AbA. The following step and α-X-gal staining were described in the yeast protocol handbook (Clontech, PT3024-1).

### Protein Extraction and Western Blot

The full-length coding sequences of *SmGRAS1* were cloned into the *pMAL-2A* vector (Novagen) by using specific primers (**Table S1**). The plasmids were transformed and expressed in *Escherichia coli* cells (*Rosetta* strain). Protein induction and purification were performed as previously described (Bai et al., 2018). The SDS-PAGE analyses of MBP (malE) and SmGRAS1-MBP purified proteins were conducted and showed major bands with an approximate molecular mass of 96.4 kDa (**Figure S6**). Subsequently, the purified proteins were verified using western blot as previously described (Ru et al., 2017).

### Dual-Luciferase Assay

The 649-bp promoter of *SmKSL1* was cloned and inserted into *pGREEN*. The vector *pCAMBIA1304-SmGRAS1* was transferred into *Agrobacterium* strain GV3101. *pCAMBIA1304* empty vector was used as a negative control and the 35S promoter-driven Renilla luciferase as an internal control. The two GV3101 strains were co-infiltrated into tobacco leaves. Infiltrated leaves were incubated in darkness for 8 h and then in light for 40 h. Three biological replicates of each sample were assayed using the Dual-Luciferase Reporter Assay System (Promega, USA).

### EMSA Analysis

The oligonucleotide probes were synthesized (listed in **Table S1**) and annealed at 95°C for 5 min, followed by cooling to room temperature. EMSA was performed using the EMSA kit (Invitrogen, USA), and a protein-free sample was used as the blank control. The mass ratios of the probe and protein were 1:5/15/50 in each reaction mixture (10 μl). The gels were imaged on a 490 nm SYBR photographic filter using a ChemiDoc XRS+ system (Bio-Rad, USA).

### Y2H Assays

The full-length coding sequences of *SmGRAS1* were inserted into the *pGADT7* vector and SmGRAS2 were inserted into the *pGBKT7* vector by using specific primers (**Table S1**). The *SmGRAS1-AD* and *SmGRAS2-BD* plasmids were co-transformed into strain Y2H. The *pGBKT7* and *pGADT7* vectors were co-transformed to serve as a negative control. After selection on SD/-Leu/-Trp, single transformant colonies were screened for growth on a SD/-Ade/-His/-Leu/-Trp with AbA and α-X-gal. Interactions were observed after 3-day incubation at 29°C.

## Results

### Characterization of SmGRAS1/2

To study the functions of *SmGRAS* genes, their coding regions were amplified to generate transgenic hairy roots. We first obtained two *SmGRAS* genes (*SmGRAS1/2*) transgenic hairy roots. The ORFs of *SmGRAS1* (GenBank accession number KY435886) and *SmGRAS2* (GenBank accession number KY435887) encode 489 and 459 amino acids, respectively. Phylogenetic analysis indicated that SmGRAS1 clustered with *Arabidopsis* AtSHR, while SmGRAS2 clustered with AtPAT1 (**Figure S1**). The SHR and PAT1 subfamilies are involved in the root development, light signaling, and stress tolerance. The results indicated the potential functions of the two genes in the root development of *S. miltiorrhiza*.

### Expression Pattern of SmGRAS1/2

**Figure 1:**
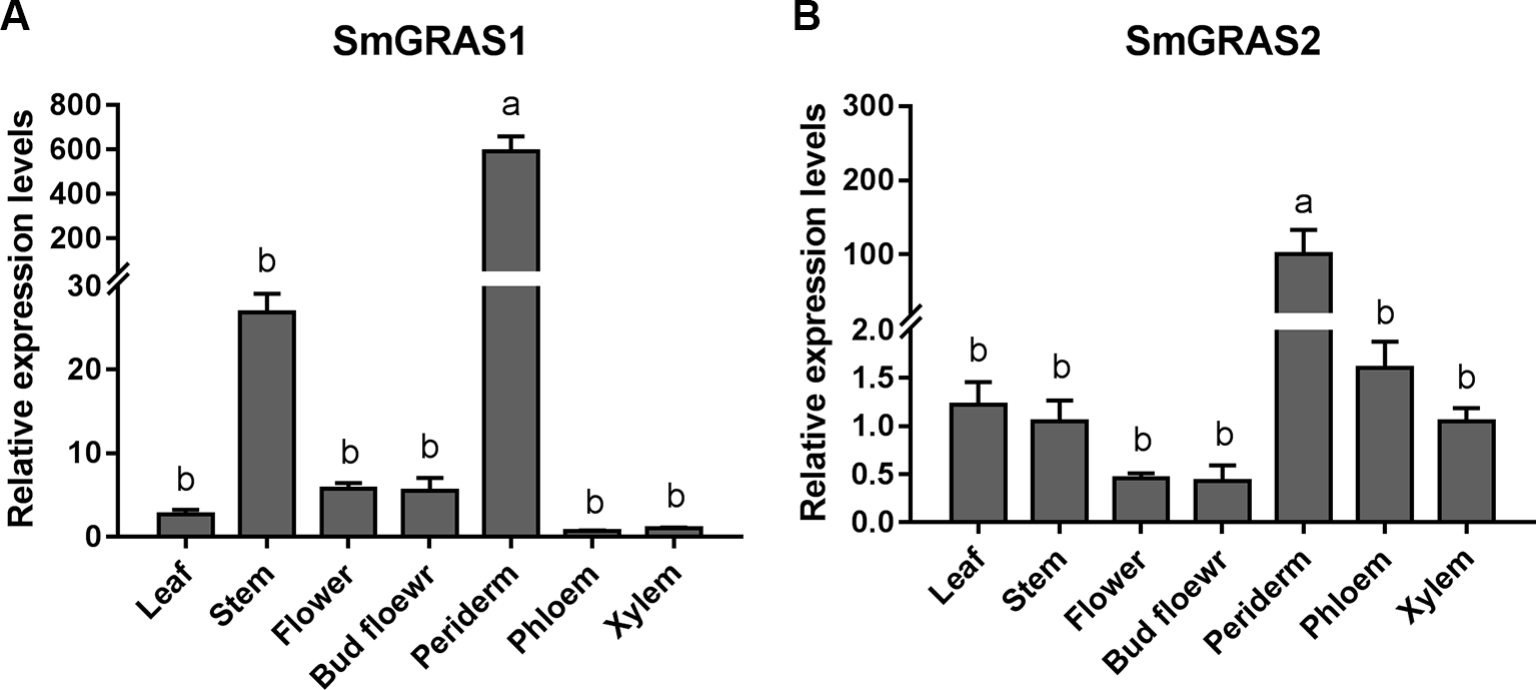
Tissue-specific expressions of *SmGRAS1* and *SmGRAS2*. **(A)** Tissue-specific expressions of *SmGRAS1* in leaf, stem, flower, bud flower, periderm, phloem, and xylem tissues of *S. miltiorrhiza* roots. **(B)** Tissue-specific expressions of *SmGRAS2* in leaf, stem, flower, bud flower, periderm, phloem, and xylem tissues of *S. miltiorrhiza* roots. The expression levels were normalized to values in the xylem. Standard errors were calculated from three sets of biological replicates. Significant differences using one-way ANOVA and S-N-K comparison tests, *P* < 0.05.

To determine the potential functions of *SmGRAS1* and *SmGRAS2* in *S. miltiorrhiza*, we detected their expression patterns in leaf, stem, flower, bud flower, periderm, phloem, and xylem tissue (**Figure 1**). *SmGRAS1* and *SmGRAS2* were expressed in all these tissues, remarkably higher in the periderm. Considering that diterpenoid tanshinones not only accumulate but are also biosynthesized in the periderm of *S. miltiorrhiza* roots (Xu et al., 2015a; Xu et al., 2015b), the highest expressions of *SmGRAS1* and *SmGRAS2* in the periderm suggest that *SmGRAS1/2* might be functionally involved in tanshinones biosynthesis in the periderm of *S. miltiorrhiza* roots.

### Subcellular Localization and Transactivation Activity of SmGRAS1/2

**Figure 2:**
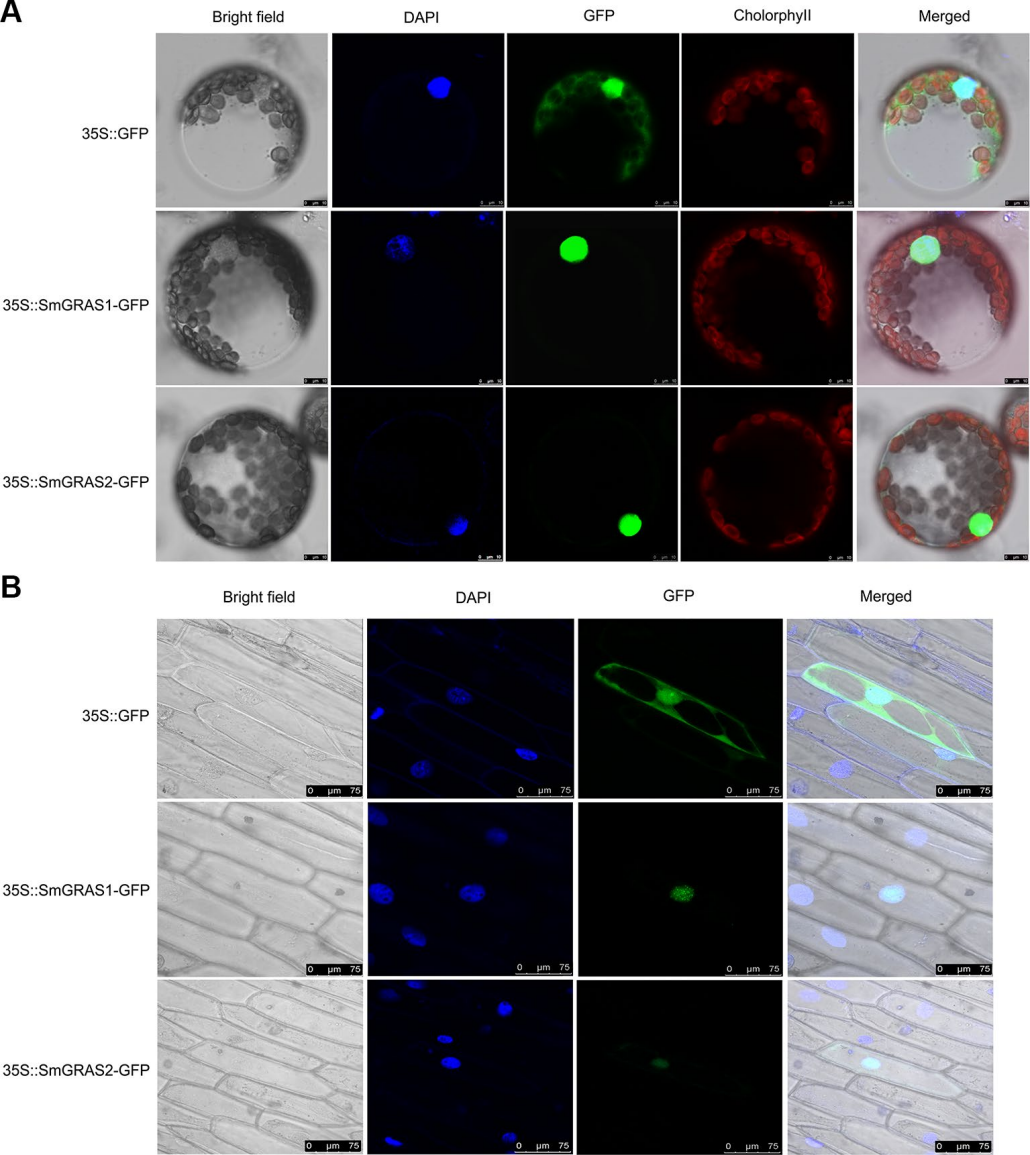
Subcellular localization of SmGRAS1 and SmGRAS2. **(A)** Subcellular localization of SmGRAS1 and SmGRAS2 in protoplasts of tobacco leaves. **(B)** Subcellular localization of SmGRAS1 and SmGRAS2 in onion epidermal cells. Upper images represent the green fluorescent protein (GFP) control, while lower images represent the SmGRAS1/2-GFP fusion proteins. GFP, green fluorescence; DAPI, fluorescence of DAPI nuclear dye; Cholorophyll, chloroplast autofluorescence; Bright field, field observations; Merged, merge of bright field and relevant fluorescence.

To identify the subcellular localization of SmGRAS1/2, we fused the SmGRAS1/2 proteins with GFP label. The GFP signal was scanned in the protoplasts of tobacco leaves (**Figure 2A**) and onion epidermal cells (**Figure 2B**). The results showed that the GFP controls distributed throughout the cell, while the SmGRAS1/2 were localized only in the nucleus. The results indicated that SmGRAS1 and SmGRAS2 might function as TF.

To further verify the characteristics of SmGRAS1/2, the transactivation activity of SmGRAS1/2 were analyzed. The results showed that the *SmGRAS1/2-pGBKT7* and control yeast were able to survive on SD/-Trp medium. The yeast with *SmGRAS1-pGBKT7* and positive control grew normally but the yeast with *SmGRAS2-pGBKT7* and negative control constructs could not grow on SD/-Trp-His-Ade medium (**Figure S2**). These results demonstrated that SmGRAS1 had transcriptional activity, while SmGRAS2 had no transcriptional activity.

### SmGRAS1/2 Regulate the Root Growth and Biosynthesis of Tanshinones, GA, and Phenolic Acids

**Figure 3:**
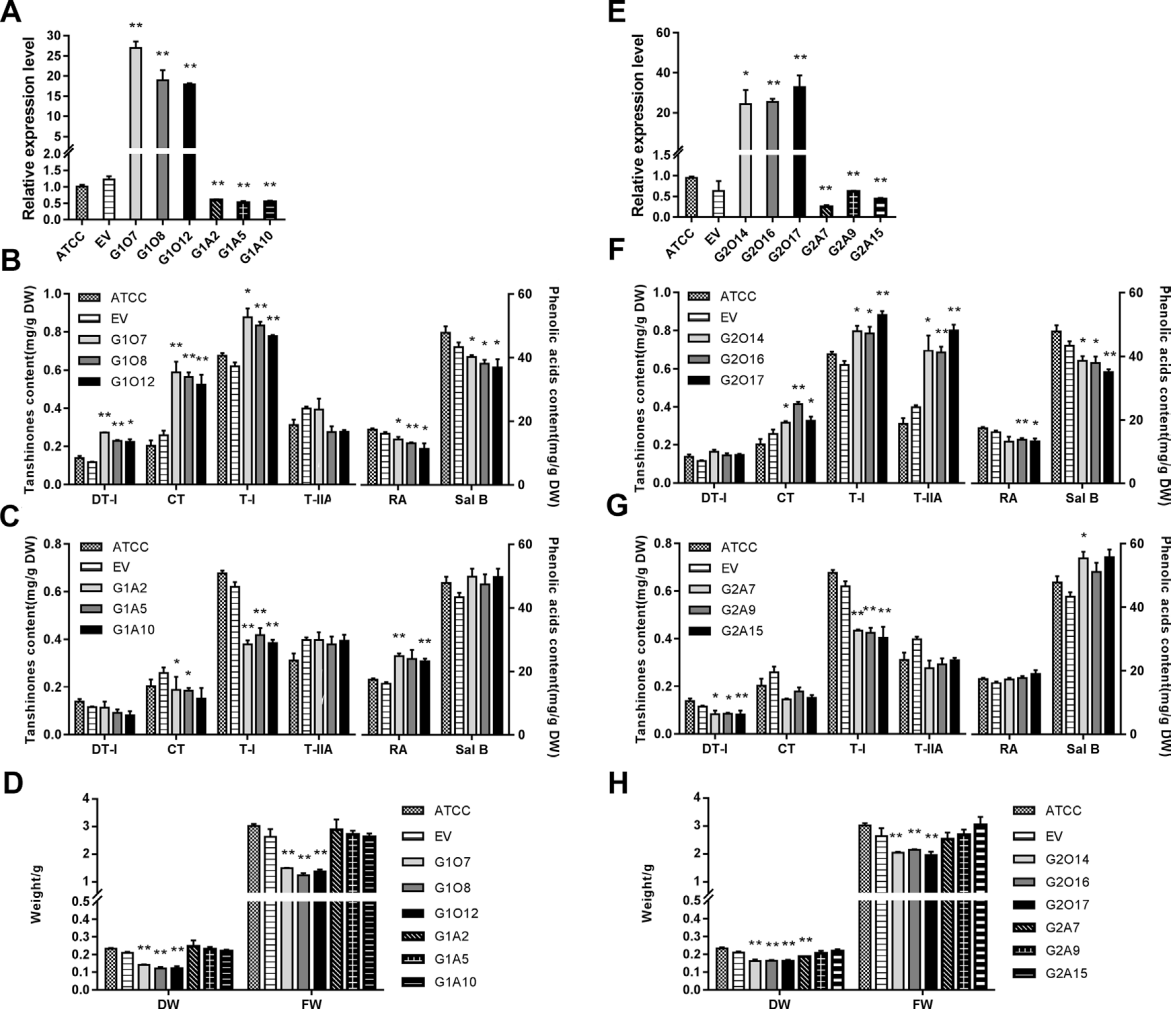
Root growth and the biosynthesis of tanshinones, phenolic acids, and GA in the control and transgenic hairy roots. **(A**, **E)** Relative quantitative analysis of *SmGRAS1* and *SmGRAS2* expressions in the transgenic lines and controls. **(B**, **C)** Analysis of tanshinones and phenolic acids productions from *SmGRAS1* OE and AE hairy root lines. **(F**, **G)** Analysis of tanshinones and phenolic acids productions from *SmGRAS2* overexpression (OE) and antisense expression (AE) hairy root lines. **(D**, **H)** Root biomass of *SmGRAS1* and *SmGRAS2* transgenic lines. Standard errors were calculated from three sets of biological replicates. Significant differences using Student’s t-test, * 0.01 < *P* < 0.05, ** *P* < 0.01).

To explore the regulatory role of SmGRAS1/2 in the biosynthesis of tanshinones, phenolic acids, and GA, OE and antisense expression (AE) approaches were used to generate respective transgenic hairy roots lines. The positive transgenic hairy roots were identified by PCR (**Figure S3**). Hairy roots developed using *ATCC15834* without plasmids were the controls (ATCC). Three independent OE and AE lines of each gene were selected for further experiments. Together, the expression of *SmGRAS1/2* were 15–35 fold higher in the OE lines than in the control but decreased by 30%–70% in the AE lines (**Figures 3A, E**).

The biomass of the *SmGRAS1/2* OE lines was significantly reduced, and that of the AE lines showed little change compared with the control (**Figures 3D, H**), which could be due to the redundancy among the *SmGRAS* genes. This result was consistent with the phenotypes of the control and *SmGRAS1/2* transgenic hairy roots (**Figure S4**). The results indicated that SmGRAS1/2 could inhibit the root growth.

The HPLC analysis showed that the tanshinones (DT-I, CT, T-I, T-IIA) contents were significantly increased in the *SmGRAS1/2* OE lines compared to the controls (**Figures 3B, F**). CT content in *SmGRAS1* OE lines and T-IIA content in *SmGRAS2* OE lines increased the most, reaching about 2-fold of the controls. In contrast, the contents of four tanshinones were reduced in the *SmGRAS1/2 AE* lines, especially T-I (**Figures 3C, G**). In addition, the RA and salvianolic acid B contents were decreased in the *SmGRAS1/2* OE lines and increased in the AE lines (**Figures 3B, C, F, G**). The results showed that SmGRAS1/2 promoted the accumulation of tanshinones but reduced the accumulation of phenolic acids. As we speculated that the decline of root biomass in the *SmGRAS1/2* OE lines might be associated with the decrease of active GA content, we also quantified the GA content in the G1O7 and G2O17 lines. The concentration of GA in the *SmGRAS1/2* OE lines was significantly decreased by more than a half compared to that of the control lines (**Figure 5G**). These data supported the speculation that the inhibition of root growth was tightly associated with a reduced GA content.

**Figure 4:**
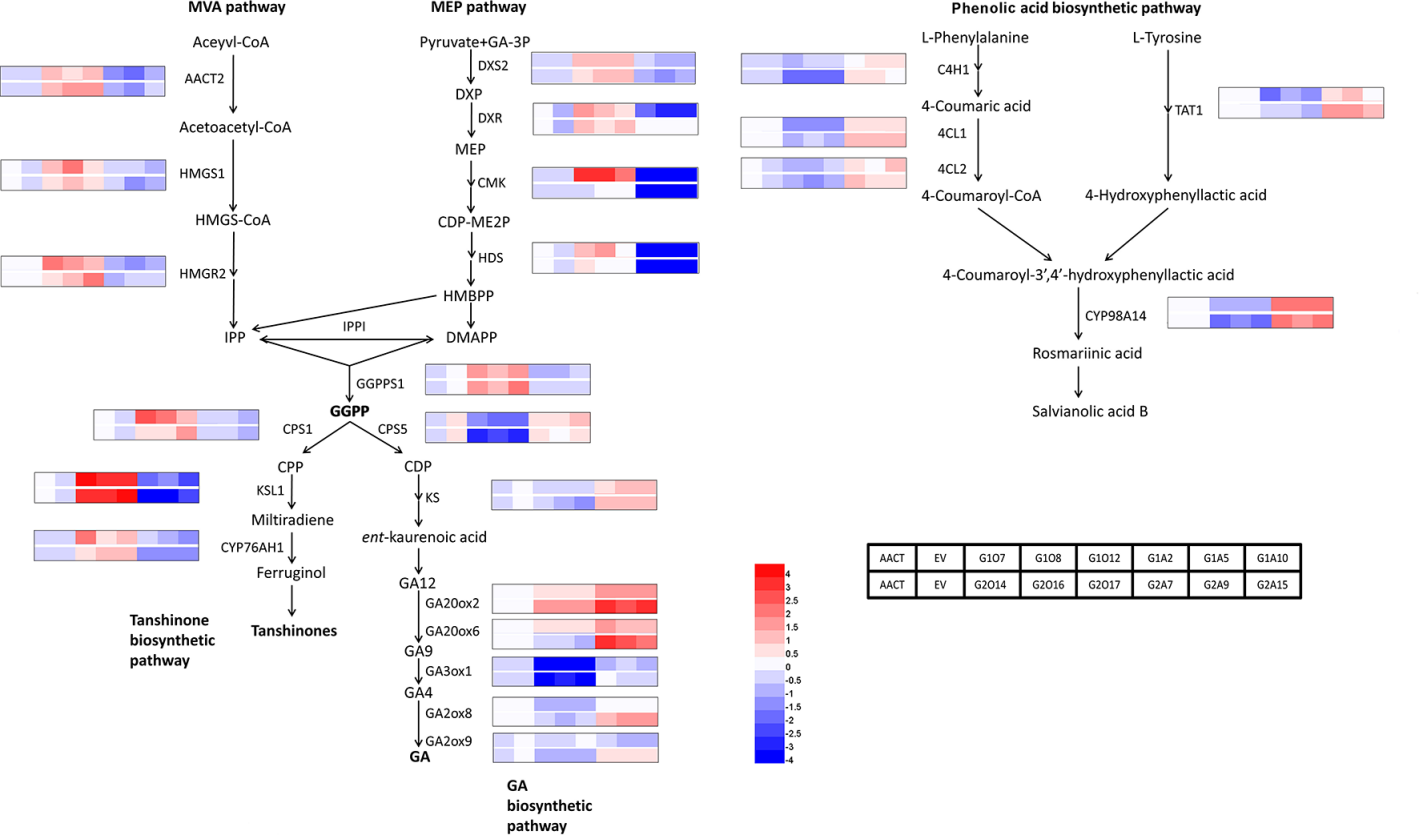
Relative expression levels of tanshinones, phenolic acids and GA biosynthesis pathway genes in *SmGRAS1/2* transgenic and control hairy roots (21-day-old). Each line had three biological replicates. The expression levels were normalized to values in the ATCC lines. Blocks with colors indicate low/downregulated expression (blue), high/upregulated expression (red), or no expression/no change (white).

To identify biosynthetic genes regulated by SmGRAS1/2 in *S. miltiorrhiza*, we measured the expressions of key enzyme genes in the tanshinones, GA, and phenolic acids biosynthetic pathways (**Figure 4**). As expected, expressions of the genes whose promoters contained the GA response element GARE motif and P-box were consistent with HPLC results. Expressions of most tanshinones biosynthetic genes, except for *CMK* and *HDS*, were upregulated to various degrees in the *SmGRAS1/2* OE lines. Among these genes, the first key enzyme gene *CPS1* in the biosynthesis from diterpenoids common precursor GGPP to tanshinones was upregulated and the downstream gene *KSL1* was the most dramatically upregulated one in the *SmGRAS1/2* OE lines. In contrast, expressions of most tanshinones biosynthetic genes were decreased in the AE lines. Expressions of most of GA biosynthetic downstream genes, except for *GA20ox2/6,* were inhibited in the *SmGRAS1/2* OE lines. And the first key enzyme gene *CPS5* in the biosynthesis from diterpenoids common precursor GGPP to GA was downregulated in the *SmGRAS1/2* OE lines. In addition, the expressions of most phenolic acids biosynthetic genes were downregulated in the *SmGRAS1/2* OE lines and upregulated in the AE lines. Collectively, our data indicated that SmGRAS1/2 could regulate the biosynthesis of tanshinones, phenolic acids, and GA through regulating the expressions of key biosynthesis genes. Taken together, our results suggested that SmGRAS1/2 inhibited root growth, GA and phenolic acids biosynthesis, but promoted tanshinones biosynthesis.

### Roles of SmGRAS1/2 in the GA-Mediated Root Growth and Biosynthesis of Tanshinones and Phenolic Acids

**Figure 5:**
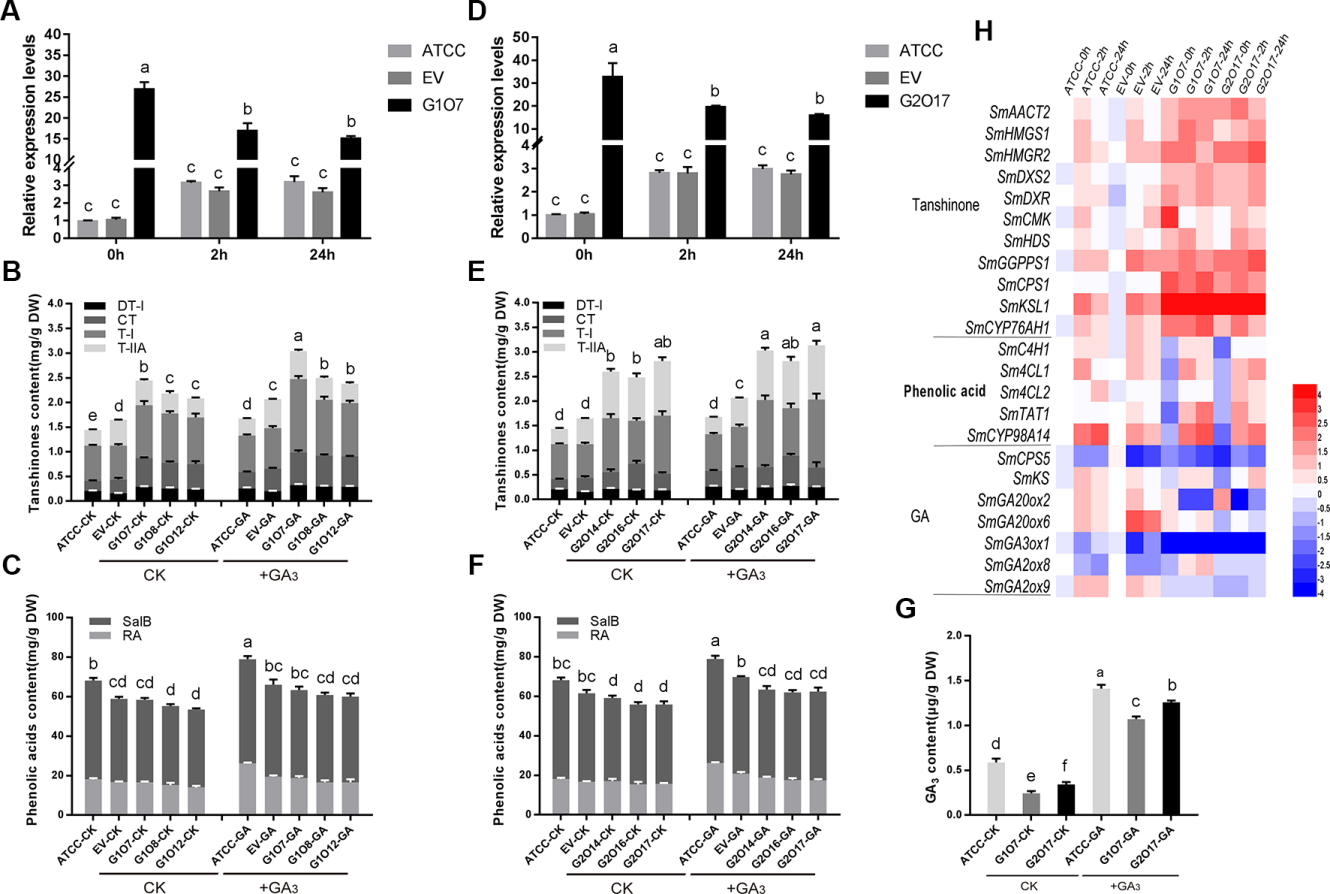
GA affects the root biomass and the contents of tanshinones, phenolic acids, and GA in *SmGRAS1* and *SmGRAS2* OE hairy roots. **(A**, **D)** Relative quantitative analysis the expressions of *SmGRAS1* and *SmGRAS2* in the OE lines and controls with or without 100 µM GA treatment for 2/24 h. **(B**, **C)** Analysis of tanshinones and phenolic acids productions from *SmGRAS1* OE lines and controls with or without 100 µM GA treatment for 6 days. **(E**, **F)** Analysis of tanshinones and phenolic acids productions from *SmGRAS2* OE lines and controls with or without 100 µM GA treatment for 6 days. **(G)** Analysis of GA production from *SmGRAS1/2* OE lines and controls with or without 100 µM GA treatment for 6 days. **(H)** Relative expression levels of genes involved in tanshinones, phenolic acids and GA biosyntheses in the *SmGRAS1/2* OE lines with 100 µM GA treatment for 2/24 h. Standard errors were calculated from three sets of biological replicates. Significant differences using one-way ANOVA and S-N-K comparison tests, *P* < 0.05.

Since overexpressing of *SmGRAS1/2* caused the transgenic hairy roots to grow slower, the inhibition of root growth was similar to the GA-deficient phenotypes. To further confirm whether the regulatory functions of SmGRAS1/2 are GA-dependent, we then used GA to treat the *SmGRAS1/2* OE and control lines. GA treatment significantly upregulated and downregulated the expressions of *SmGRAS1/2* in the control lines and *SmGRAS1/2* OE lines, respectively (**Figures 5A, D**). The root biomass and GA content of *SmGRAS1/2* OE lines were significantly increased under GA treatments (**Figures 5G**, **S5**), which showed that the inhibition of SmGRAS1/2 in root growth might be mainly caused by GA deficiency. Intriguingly, tanshinones contents were also significantly increased under GA treatments (**Figures 5B, E**). Furthermore, GA treatment increased the phenolic acids contents in the control and *SmGRAS1/2* OE lines (**Figures 5C, F**). Collectively, these changes in root biomass, GA, and phenolic acids contents in the *SmGRAS1/2* OE lines were the opposite after GA treatment. These results indicated that SmGRAS1/2 played negative roles in GA-regulated root growth and phenolic acids biosynthesis but that the roles of SmGRAS1/2 in regulating tanshinones biosynthesis were an exception.

As expected, the expressions of most tanshinones biosynthesis genes were quickly induced by GA application, as shown by an early peak at 2 h and a relatively high level of expressions at all sampling times (**Figure 5H**). Among these genes, the expression of downstream key enzyme gene *KSL1* had the most significant increasement. Expressions of most phenolic acids biosynthesis genes were also upregulated. The expressions of phenolic acids biosynthesis genes and downstream key enzyme gene *CYP98A14* were significantly increased. In addition, the expressions of GA biosynthesis key genes were different and *GA30xo1* was the most significantly downregulated gene among them. Collectively, the expressions of these biosynthetic pathway genes were consistent with the content changes. Taken together, our results indicated that SmGRAS1/2 regulated root growth and phenolic acids biosynthesis probably through GA-dependent pathways but the regulation of tanshinones biosynthesis was not.

### SmGRAS1 Binds to the Promoter of *SmKSL1* Involved in Tanshinones Biosynthesis

**Figure 6:**
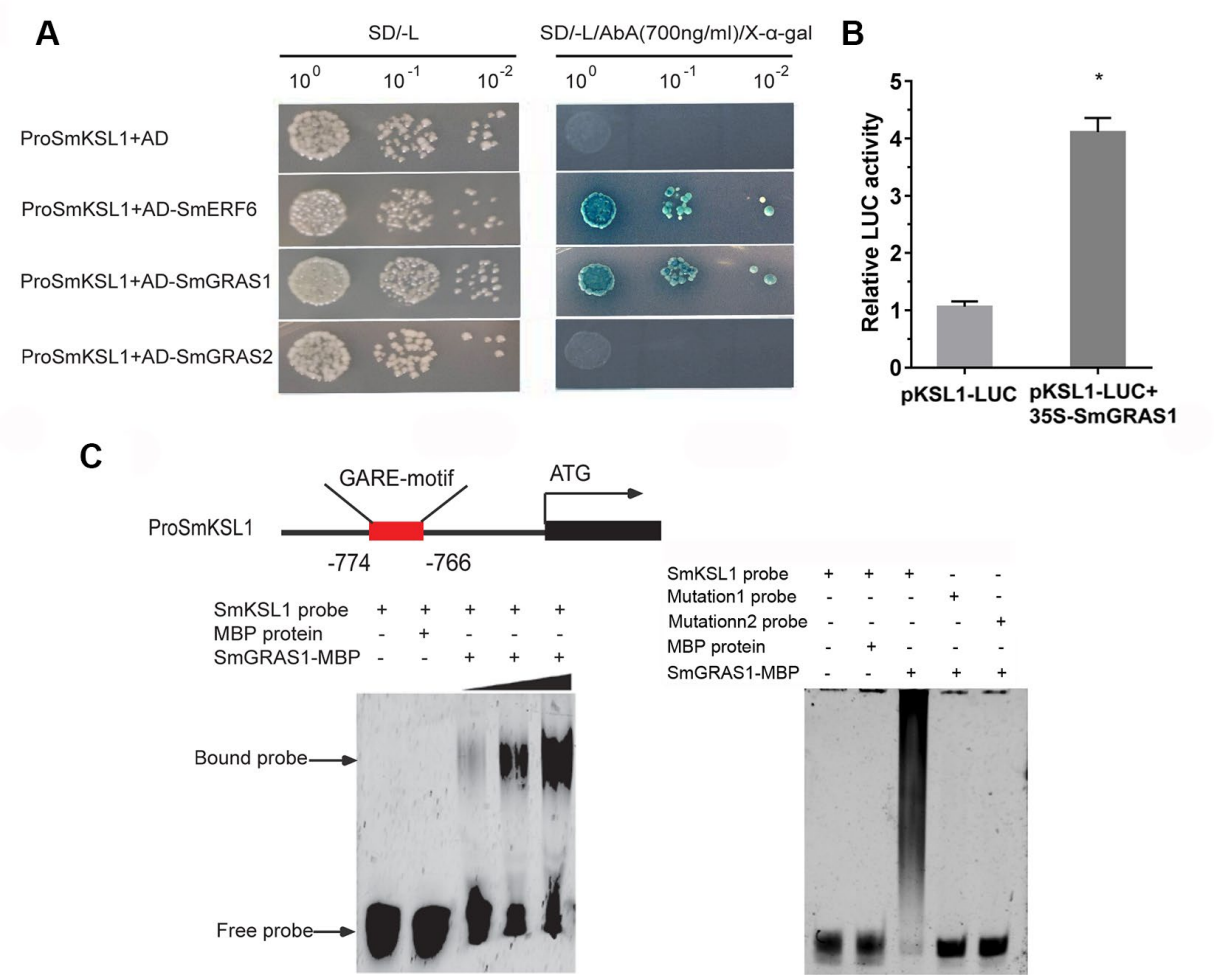
SmGRAS1 binds to the *SmKSL1* promoter and activates its expression. **(A)** Yeast one-hybrid (Y1H) assays shows the interactions between SmGRAS1/2 and the *SmKSL1* promoter. *SmKSL1* promoter + *pGADT7* as the negative control and *SmKSL1* promoter + *pGADT7-SmERF6* as the positive control. **(B)** Dual-luciferase (Dual-LUC) assay shows the effects of SmGRAS1 on *SmKSL1* promoter activation. **(C)** Electrophoretic mobility shift assay (EMSA) analysis of SmGRAS1 binding to the GARE-motif of the *SmKSL1* promoter. Schematic diagram showing the GARE motif in the *SmKSL1* promoter.

Because only the regulation of SmGRAS1/2 to tanshinones biosynthesis was not affected by GA treatment, we speculated that SmGRAS1/2 might directly regulate the expressions of the tanshinones biosynthetic pathway genes. According to our qRT-PCR results, *SmKSL1*, which is the key downstream gene in tanshinones biosynthesis, was remarkably upregulated in *SmGRAS1/2* OE lines. Moreover, its promoter had a GA response elements GARE motif. Y1H and EMSA assays were performed to demonstrate whether SmGRAS1/2 could bind to the GARE motif of the *SmKSL1* promoter. The Y1HGold reporter of the strains that had the *SmKSL1* promoter transformed with SmGRAS1 prey plasmid could grow on SD/-Leu (700 ng/ml AbA) but the strains that had the SmGRAS2 prey plasmid could not grow (**Figure 6A**). These results showed that SmGRAS1 could directly bind to *SmKSL1* promoter. This was further confirmed by Dual-LUC report system. Dual-LUC assay showed that SmGRAS1 could directly activate the *SmKSL1* promoter (**Figure 6B**).

To further confirm the bond between SmGRAS1 and the GARE motif of the *SmKSL1* promoter *in vitro*, purified SmGRAS1-MBP fusion proteins were combined with the fragment containing the GARE motif and they were analyzed by EMSA. Subsequently, specific DNA–SmGRAS1 protein complex was strongly detected (**Figure 6C**). However, SmGRAS1 could not bind to the mutated GARE motif fragments (**Figure 6C**). These results confirmed that SmGRAS1 participated in regulating tanshinones biosynthesis by directly binding to the GARE motif of the *SmKSL1* promoter.

### Physical Interaction Between SmGRAS1 and SmGRAS2

**Figure 7:**
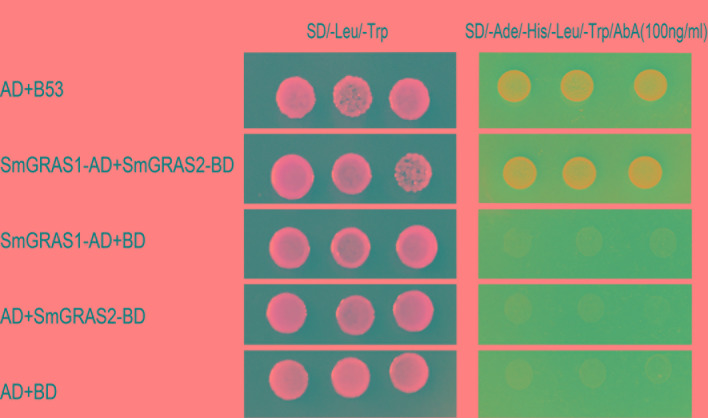
SmGRAS1 interacts with SmGRAS2. Yeast two-hybrid (Y2H) assays to detect the interaction of SmGRAS1 with SmGRAS2. Transformed Y2H are grown on SD/-Leu/-Trp or SD/-Ade/-His/-Leu/-Trp/AbA with α-X-Gal. The co-transformed *pGBKT7* and *pGADT7-53* vector as the positive control, *pGBKT7* and *pGADT7-lam* vector as the negative control.

Since SmGRAS1 and SmGRAS2 had similar functions in regulating the biosynthesis of tanshinones and phenolic acids, Y2H assays were utilized to investigate this interaction. Y2H yeast cells co-transformed by SmGRAS1-AD and SmGRAS2-BD not only grew well on SD/-Leu/-Trp medium, but also grew on the SD/-Ade/-His/-Leu/-Trp/AbA medium, and could turn blue in the α-X-Gal staining assay. However, all the Y2H yeast cells that harbored the negative controls could only grow on the SD/-Leu/-Trp medium but not on SD/-Ade/-His/-Leu/-Trp/AbA medium (**Figure 7**).

## Discussion

### SmGRAS1/2 Involved in Tanshinones Biosynthesis

As the major active ingredient of *S. miltiorrhiza*, tanshinones contents have been reported to be concentrated in the periderm of root (Xu et al., 2015a; Xu et al., 2015b). As the key regulators in GA signal, GRAS family genes have been reported involved in the regulation of root growth (Gong et al., 2016). Phylogenetic analysis indicated that SmGRAS1 clustered with *Arabidopsis* AtSHR, which was highly expressed in root tip tissue and participated in *Arabidopsis* root development (Cui et al., 2007). SmGRAS2 clustered with AtPAT1, which had been reported to participate in light signaling and stress tolerance (Hakoshima, 2018). The tissue-specific expressions of *GRAS* genes pointed to their functional roles in root development. For instance, *VviSHR3* was more highly expressed in the roots than in other tissues in *Vitis vinifera*, and its tomato ortholog *SlGRAS16* was also predicted to be involved in root development (Huang et al., 2015; Grimplet et al., 2016). Considering that diterpenoids tanshinones not only accumulate but are also biosynthesized in the periderm of *S. miltiorrhiza* roots (Xu et al., 2015a; Xu et al., 2015b), the highest expressions of *SmGRAS1* and *SmGRAS2* in the periderm indicated that *SmGRAS1/2* functionally involved in tanshinones biosynthesis in the periderm of *S. miltiorrhiza* roots. Moreover, GA could increase tanshinones accumulation and induce the *SmGRAS1* and *SmGRAS2* genes response in the wild-type hairy roots of *S. miltiorrhiza* in our previous study (Bai et al., 2017), which further confirmed the potential functions of two genes in GA-mediated secondary metabolite accumulation in the roots of *S. miltiorrhiza*.

### SmGRAS1/2 Promote Tanshinones Biosynthesis and Inhibit GA Biosynthesis by Regulating the Metabolic Flux in the Roots of *S. miltiorrhiza*

Both as diterpenoids, GA and tanshinones has a common biosynthesis precursor GGPP. There exists a tradeoff between GA and tanshinones biosynthesis. The biosynthetic pathways of GA and tanshinones involves many enzymes. Many TFs have been reported to have universal regulatory functions in terpenoid biosynthesis (Lu et al., 2016). However, studies on the regulation of SmGRASs to secondary metabolism in *S. miltiorrhiza* have not been reported. In our study, *SmGRAS1/2* OE hairy roots grow slower than the control. Similarly, tomato primary and lateral root growth in *SlGRAS24* OE lines were strongly suppressed (Huang et al., 2017). The results indicated that SmGRAS1/2 were the inhibitors of root growth. And the GA content in the OE lines was also decreased higher than the control. These data supported the speculation that the inhibition of root growth was tightly associated with a reduced GA content. It has been reported that overexpressing *HaGRASL* reduces the metabolic flow of GAs in *Arabidopsis*, which could be relevant in axillary meristem development (Fambrini et al., 2015). Silencing *SlGRAS26* inhibit the GA biosynthetic pathway but promote the GA inactivation pathway, and finally resulted in GA deficiency in tomato (Zhou et al., 2018). The decreased expression of *SlGRAS2* is associated with a reduction in active GA, leading to a deficiency in positive growth signals during ovary development in tomato (Li et al., 2018). Overexpressing of *SmGRAS1/2* also inhibited the phenolic acids and promoted tanshinones biosynthesis through regulating the biosynthetic pathway genes. Many biosynthetic pathway genes such as *C4H, TAT, DXS, GGPPS, and CYP76AH1*, have been reported to promote the phenolic acids or tanshinones accumulation (Xiao et al., 2011; Ma et al., 2016; Shi et al., 2016). The downstream pathway genes from GGPP to GA were downregulated and the genes from GGPP to tanshinones was upregulated. Therefore, these results implied that SmGRAS1/2 could regulate the flow of metabolites by catalyzing the precursor GGPP to synthesize more tanshinones but inhibit the GA biosynthetic pathway. Taken together, SmGRAS1/2 acted as positive regulators of tanshinones biosynthesis and negative regulators of GA and phenolic acids biosynthesis.

### SmGRAS1/2 Regulate Root Growth and Phenolic Acids Biosynthesis in GA-Dependent Pathway, But Regulate Tanshinones by Directly Binding to the *SmKSL1* Promoter

Since *SmGRAS1/2* OE lines showed some GA-deficient phenotypes. To explore whether the regulations of SmGRAS1/2 to phenolic acids and tanshinones biosynthesis were involved in GA signaling pathway, we treated *SmGRAS1/2* OE and control lines with GA. GA treatment recovered the inhibition of SmGRAS1/2 on root growth. And the GA content was also increased after GA treatment. The result showed that the increased GA content in the *SmGRAS1/2* OE lines maybe not have occurred by promoting GA biosynthesis but could have also been caused by exogenous GA entering the cell. And the inhibition of SmGRAS1/2 in root growth was mainly caused by the GA deficiency, and the inhibition could be restored by adding GA.

In addition, GA has been reported to promote the accumulation of secondary metabolites as well as the expressions of related biosynthetic genes. For instance, GA could induce *SmHPPR* response, which was highly correlated with hydrophilic phenolic acids accumulation (Wang et al., 2017). After GA treatment, the phenolic acids contents were increased and most of the biosynthetic pathway genes were also upregulated, which had opposite changes compared to the OE lines before treatment. The results showed that SmGRAS1/2 regulated phenolic acids biosynthesis dependent on GA signaling pathway. SmGRAS1/2 played negative regulatory roles in GA-mediated root growth and phenolic acids biosynthesis.

Interestingly, the tanshinones contents of *SmGRAS1/2* OE and control lines all increased after GA treatment. This result indicated that SmGRAS1/2 and GA had some independent ways of regulating tanshinones biosynthesis. Studies had shown that GRAS could interact with the promoter of downstream genes and regulate their expressions (Hirsch et al., 2009). For instance, OsGRAS23 could bind to the promoters of its potential target genes to positively modulate rice drought tolerance (Xu et al., 2015a; Xu et al., 2015b). SlGRAS2 regulated the expressions of downstream genes related to fruit development (Li et al., 2018). We speculated that SmGRAS1/2 could directly regulated tanshinones biosynthesis genes. Considering the significant response of *SmKSL1* to SmGRAS1/2 and GA. Y1H, Dual-LUC and EMSA assays demonstrated that SmGRAS1 could directly regulate tanshinones biosynthesis by activating *SmKSL1* rather than through GA-dependent regulation, while SmGRAS2 might regulate the tanshinones biosynthesis through interacting with SmGRAS1. These results indicated that SmGRAS1/2 played negative roles in GA-regulated root growth and phenolic acids biosynthesis but that the roles of SmGRAS1/2 in regulating tanshinones biosynthesis were an exception.

However, *SmKSL1* was likely not the only target gene for GRAS1 regulation. GRAS could also interact with other TFs to mediate the regulation of transcription activity of other target genes. For instance, DELLA protein could interact with SG7 MYBs to regulate the transcriptional levels of the flavonol biosynthesis pathway key genes (Tan et al., 2019). Therefore, identifying new interactive partners or targets of SmGRAS1/2 may provide further insight into the molecular mechanism of SmGRAS1/2-mediated regulation of secondary metabolite biosynthesis.

## Conclusion

As the medicinal parts of *S. miltiorrhiza*, the roots contain very low contents of tanshinones and phenolic acids. Therefore, improving *S. miltiorrhiza* root biomass and the accumulation of the two major bioactive compounds, tanshinones and phenolic acids, in *S. miltiorrhiza* roots has a crucial influence on the quality of medicinal materials. However, few functional genes have been reported to regulate both root growth and secondary metabolism. Our study revealed that SmGRAS1/2 could regulate the flow of diterpenoids biosynthesis pathway by catalyzing the precursor GGPP to synthesize more tanshinones but inhibiting GA biosynthetic pathway. Notably, SmGRAS2 interacted with SmGRAS1 to form a complex, and promoted the biosynthesis of tanshinones through directly binding to the promoter of *SmKSL1*. In summary, SmGRAS1/2 acted as repressors in the regulation of GA-mediated root growth and phenolic acids biosynthesis and positive regulators in tanshinones biosynthesis. These results provided theoretical guidance for improving the yield and quality of medicinal materials. More work is needed to fully understand the specific mechanism of SmGRAS proteins regulate secondary metabolism in *S. miltiorrhiza*.

## Data Availability Statement

The datasets generated for this study are available on request to the corresponding author.

## Author Contributions

WL and ZL conceived and designed the experiments. WL, ZB, TP, RM, BZ, and CL preformed the experiments. WL and DY analyzed the data and wrote the manuscript. All authors read and approved the manuscript.

## Funding

This work was supported by the National Natural Science Foundation of China (No. 81373908) and the Ministry of Science and Technology of the People’s Republic of China through the Twelfth Five-Year National Science and Technology Pillar Program (No. 2015BAC01B03).

## Conflict of Interest

The authors declare that the research was conducted in the absence of any commercial or financial relationships that could be construed as a potential conflict of interest.
