# Supplementary material for: SmGRAS1 and SmGRAS2 Regulate the Biosynthesis of Tanshinones and Phenolic Acids in Salvia miltiorrhiza
Source: Front Plant Sci. 2019 Oct 30;10:1367. doi: 10.3389/fpls.2019.01367 (PMC6831727; doi:10.3389/fpls.2019.01367)
Supplement: Supplementary file 1 [file DataSheet_1.docx]

**ABBREVIATIONS**

| 4CL | 4-Coumaric acid CoA-ligase |
| --- | --- |
| AACT | Acetyl-CoA C-Acetyltransferase |
| C4H | Cinnamic acid 4-Hydroxylase |
| CMK | 4-cytidine 5-diphospho-2-C-methyl-D-erythritol kinase |
| CPS | Copalyl diphosphate synthase |
| CYP76AH1 | Cytochrome P450-dependent monooxygenase |
| CYP98A14 | Cytochrome P450-dependent monooxygenase |
| DXR | 1-Deoxy-D-xylulose 5-phosphate reductoisomerase |
| DXS | 1-Deoxy-D-xylulose 5-phosphate synthase |
| GA20ox | Gibberellin 20-oxidase |
| GA3ox | Gibberellin 3-oxidase |
| GA2ox | Gibberellin 2-oxidase |
| GGPPS | Geranylgeranyl diphosphate synthase |
| HDS | 1-Hydroxy-2-methyl-*E*-butenyl-4-diphosphate synthase |
| HMGR | 3-Hydroxy-3-methylglutaryl-CoA reductase |
| HMGS | 3-Hydroxy-3-methylglutaryl-CoA synthase |
| KS | Kaurene synthase |
| KSL | ent-Kaurene synthase-like |
| TAT | Tyrosine Amino transferase |

**TABLE S1 |** Primers used for qRT-PCR, vector construction and positive hairy roots line selection.

| Gene name | Sequence (5’ to 3’) |
| --- | --- |
| SmACT-F | GGTGCCCTGAGGTCCTGTT |
| SmACT-R | AGGAACCACCGATCCAGACA |
| SmGRAS1-F | GCCTACGACCAATCCTCCTACTCCA |
| SmGRAS1-R | CGGTCATGCGGCTGAACAATGC |
| SmGRAS2-F | ATCACCACATACGCCACGCTCTC |
| SmGRAS2-R | GCCACCAGCTTGTCGAATTCATCC |
| SmC4H1-F | TCTTGCGTTGCCTATTCT |
| SmC4H1-R | CAATGGTCGAGTGCTTCAA |
| Sm4CL1-F | ATTCGCATTCGCATTTCTCGG |
| Sm4CL1-R | GCGGCGTAGTGCTTCACCTTT |
| SmTAT1-F | AGTAGACGTGCCTGCTCT |
| SmTAT1-R | TGGCTATCCAACTCCTTC |
| SmCYP98A14-F | CCTCAACGTCGTCGTTTCCA |
| SmCYP98A14-R | AGTCCGCCCAAATCAAATCC |
| SmAACT2-F | TCACAATCTGGCTTGAAATCTTCTG |
| SmAACT2-R | AAAACAACAGCAGATGCACCTCC |
| SmHMGS1-F | TTAGGGCGAATCACATGGCTCA |
| SmHMGS1-R | TCGGCATCCAAGATCGAGAAC |
| SmHMGR2-F | GCAACATCGTCTCCGCCGTCTACA |
| SmHMGR2-R | GATGGTGGCCAGCAGCCTGGAGTT |
| SmDXS2-F | CTCACGGTCGCATTGCATCAT |
| SmDXS2-R | CGCTTTCGTCTCGTTTAGGGA |
| SmDXR-F | CATGCGTTTGCCTATTCTGTAC |
| SmDXR-R | ACTAAGAACTCCGGTCATGGTG |
| SmCMK-F | CGCTCACAATCCTAAACCACA |
| SmCMK-R | TTGAAATCCCAGCATCCCTAT |
| SmHDS-F | GTAGCCTTTCAGACCGCATTAT |
| SmHDS-R | TCCTTCCATCTTCACCCTCACC |
| SmGGPPS1-F | ACAAGACCACGTATCCCAAGC |
| SmGGPPS1-R | TCTGCCTATGTGCAATGTAATCG |
| SmCPS1-F | CCACATCGCCTTCAGGGAAGAAAT |
| SmCPS1-R | TTTATGCTCGATTTCGCTGCGATCT |
| SmKSL1-F | TGGAAACAGTGTGACCCTTCTGCT |
| SmKSL1-R | GCTTGCATACAAATAACACCCAATCCT |
| SmCYP76AH1-F | ACGCATCACTTCACCCATCTCA |
| SmCYP76AH1-R | ATTGCCGACTCATCCACGAT |
| SmKS-F | TTAGTTTTGGAGGGCAAGAAGAGTGT |
| SmKS-R | CTCCTGTTTGGTCGTTGAGAAGAATA |
| SmCPS5-F | tagaagatgcagctactttctctgct |
| SmCPS5-R | catcatcttcaccgccgtactgtt |
| SmGA20ox2-F | GATGAGCAGACTGTCGCTAGGGAT |
| SmGA20ox2-R | GCAGCTCTTGTACCTGGCGTTGGAT |
| SmGA20ox6-F | CTCCGCATTATCGAATGGAGTGT |
| SmGA20ox6-R | GGAGTGTTGCAACATCAACCCTAT |
| SmGA3ox1-F | CCGATTCGTTACTACTGACGATC |
| SmGA3ox1-R | GGACGACACGATGGCACATAGT |
| SmGA2ox8-F | CACCAACGGCAGATTCACGAGCGT |
| SmGA2ox8-R | GAGGTCGAGACGATGATCTGCCAAT |
| SmGA2ox9-F | CGACATCTTACGGGTAATGACGAATGG |
| SmGA2ox9-R | CAGCTTGCTTGTATTCACCCCAAGTG |
| SmGRAS1-GFP-F | GGGGGTCGACATGGATACTTTGTTTA |
| SmGRAS1-GFP-R | TTTTGTCGACCGAGGGTTTCCACGCACTA |
| SmGRAS1-OE-F | GGGGGCCATGGATGGATACTTTGTTTA |
| SmGRAS1-OE-R | TTTTACTAGTTCACGAGGGTTTCCAC |
| SmGRAS1-AE-F | TTTTCCATGGTCACGAGGGTTTCCAC |
| SmGRAS1-AE-R | GGGGGACTAGTATGGATACTTTGTTTA |
| SmGRAS1-AD-F | GGGGGGGGCATATGGATACTTTGTTTA |
| SmGRAS1-AD-R | TTCCCGGGTTTCACGAGGGTTTCCAC |
| SmGRAS1-BD-F | GGGGGGGGCATATGGATACTTTGTTTA |
| SmGRAS1-BD-R | TTCCCGGGTTTCACGAGGGTTTCCAC |
| SmGRAS1-MBP-F | GGGGGTCGACATGGATACTTTGTTTA |
| SmGRAS1-MBP-R | TTTTAAGCTTGCTCACGAGGGTTTCCAC |
| SmGRAS2-GFP-F | GGGGTCTAGAGATGGAATCTCATTGCT |
| SmGRAS2-GFP-R | TTTTCTAGAGCATGCCAAGCAGAAGCA |
| SmGRAS2-OE-F | GGGCCATGGATGGAATCTCATTGCT |
| SmGRAS2-OE-R | GGGACTAGTCTAATGCCAAGCAGAA |
| SmGRAS2-AE-F | CATGCCATGGCTAATGCCAAGCAGAA |
| SmGRAS2-AE-R | GGGACTAGTATGGAATCTCATTGCT |
| SmGRAS2-AD-F | GGGGGGGCATATGGAATCTCATTGCT |
| SmGRAS2-AD-R | TTCCCGGGTTCTAATGCCAAGCAGAA |
| SmGRAS2-BD-F | GGGGGGGCATATGGAATCTCATTGCT |
| SmGRAS2-BD-R | TTCCCGGGTTCTAATGCCAAGCAGAA |
| Pro-KSL1-F | GGGGGGGGGAGCTCTTCAAAATAAATAAAT |
| Pro-KSL1-R | TTTTTTCTCGAGCTTTAGCTCTGGGGCG |
| SmERF6-AD-F | GGGGGGAATTCATGATGGCAAATTCTG |
| SmERF6-AD-R | TTGAGCTCGTCAAGAAGCCGGGTTC |
| 35S-F | GACGCACAATCCCACTATCC |
| 1304GFP-R | ACCTTCACCCTCTCCACTGAC |
| rolB-F | GCTCTTGCAGTGCTAGATTT |
| rolB-R | GAAGGTGCAAGCTACCTCTC |
| rolC-F | CTCCTGACATCAAACTCGTC |
| rolC-R | TGCTTCGAGTTATGGGTACA |
| hpt II-F | CGCTTCTGCGGGCGATTTGTG |
| hpt II-R | GCTCTCGGAGGGCGAAGAATC |
| SmKSL1 probe-GARE1-F | GATTTTAGTTTAAAACAGAGCTATCGGTTTTA |
| SmKSL1 probe-GARE1-R | TAAAACCGATAGCTCTGTTTTAAACTAAAATC |
| SmKSL1 probe-Mutation1-F | GATTTTAGTTTACCCTCACGCTATCGGTTTTA |
| SmKSL1 probe-Mutation1-R | TAAAACCGATAGCGTGAGGGTAAACTAAAATC |
| SmKSL1 probe-Mutation2-F | GATTTTAGTTTAAAATCACGCTATCGGTTTTA |
| SmKSL1 probe-Mutation2-R | TAAAACCGATAGCGTGATTTTAAACTAAAATC |
| SmKSL1-Dual-LUC-F | CTGCAGGTCGACGGATCCCCGGGTTCAAAATAAATAAATCCA |
| SmKSL1-Dual-LUC-R | GGTGGACTCCTCTTAGAATTCCTTTAGCTCTGGGGCGG |


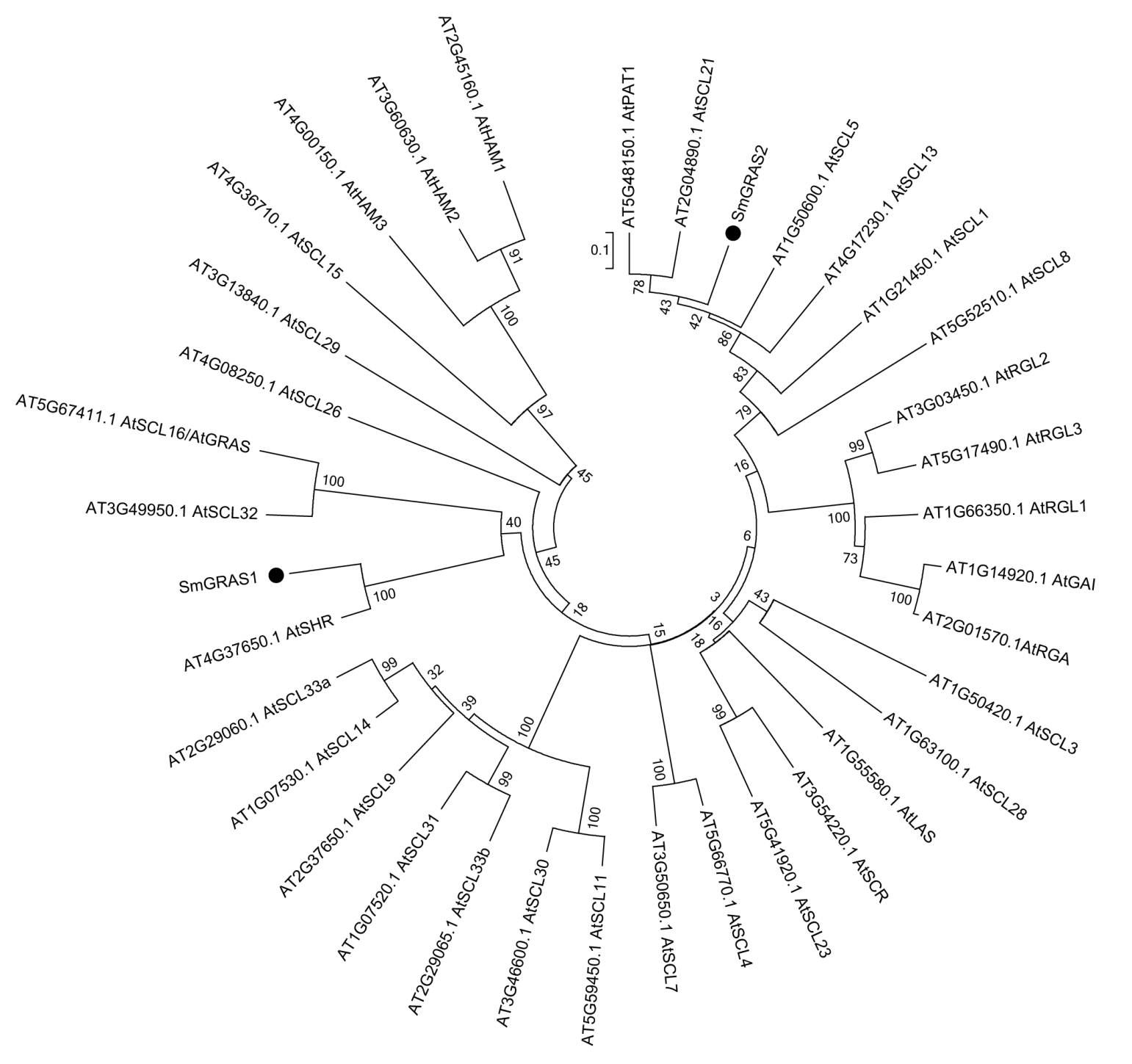


**FIGURE S1 |** Phylogenetic tree of SmGRAS1 and SmGRAS2 transcription factors. The phylogenetic tree was constructed by the neighbor-joining method of MEGA 6.0.

**
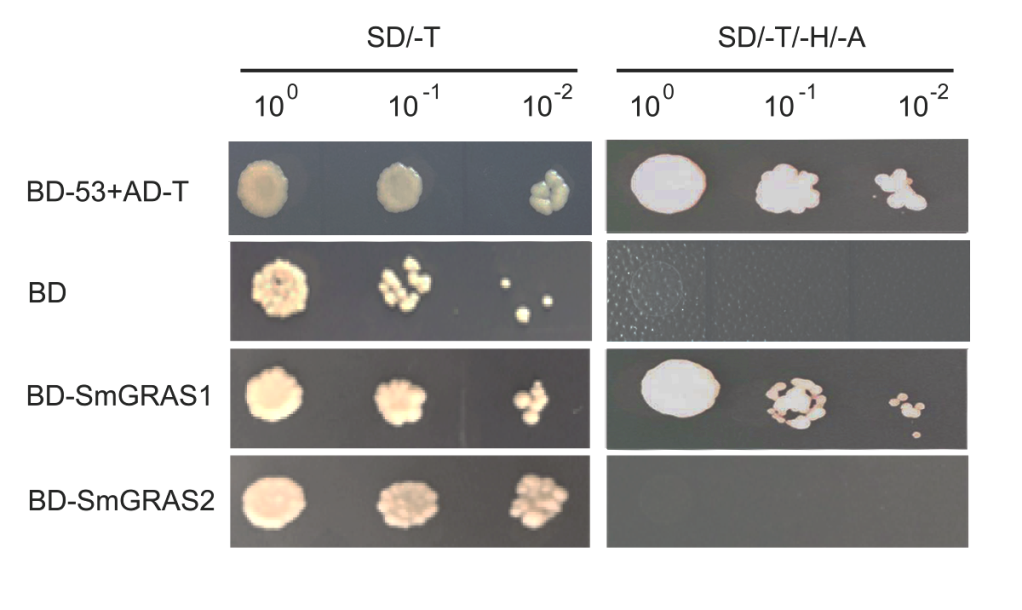
**

**FIFURE S2 |** Transactivation activity of SmGRAS1/2. Yeast AH109 containing *pGBKT7-53+pGADT7-T* (positive control), *pGBKT7* (negative control) and *SmGRAS1/2-pGBKT7* were spotted onto SD/-Trp and SD/-Trp-His-Ade medium, respectively.


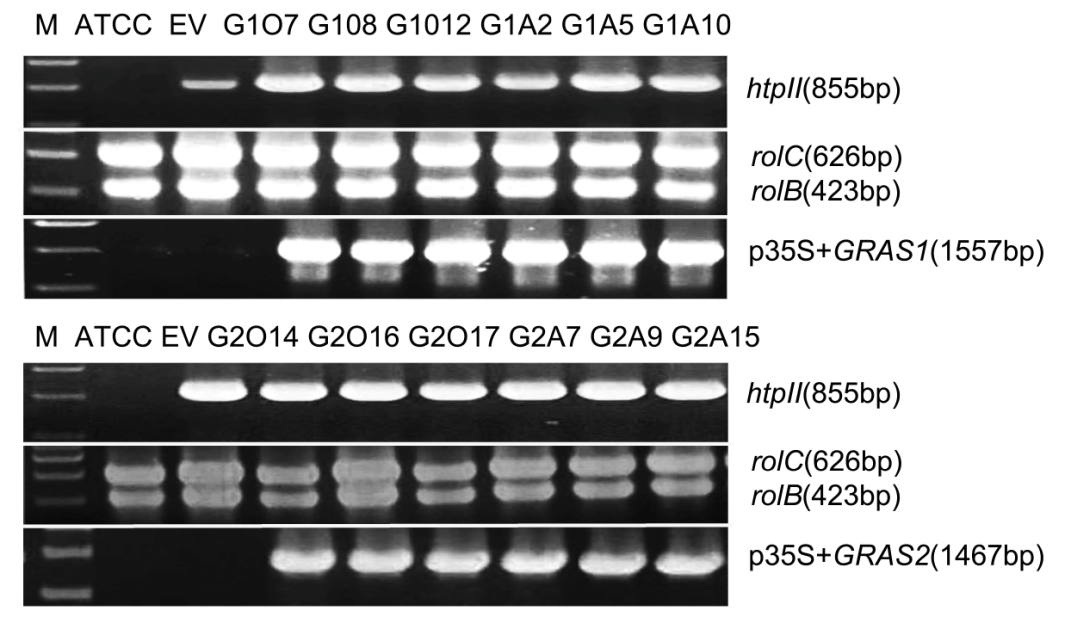


**FIGURE S3 |** Verification of positive hairy roots lines by using PCR. PCR screening of *SmGRAS1/2* overexpressing or antisense expressing lines.


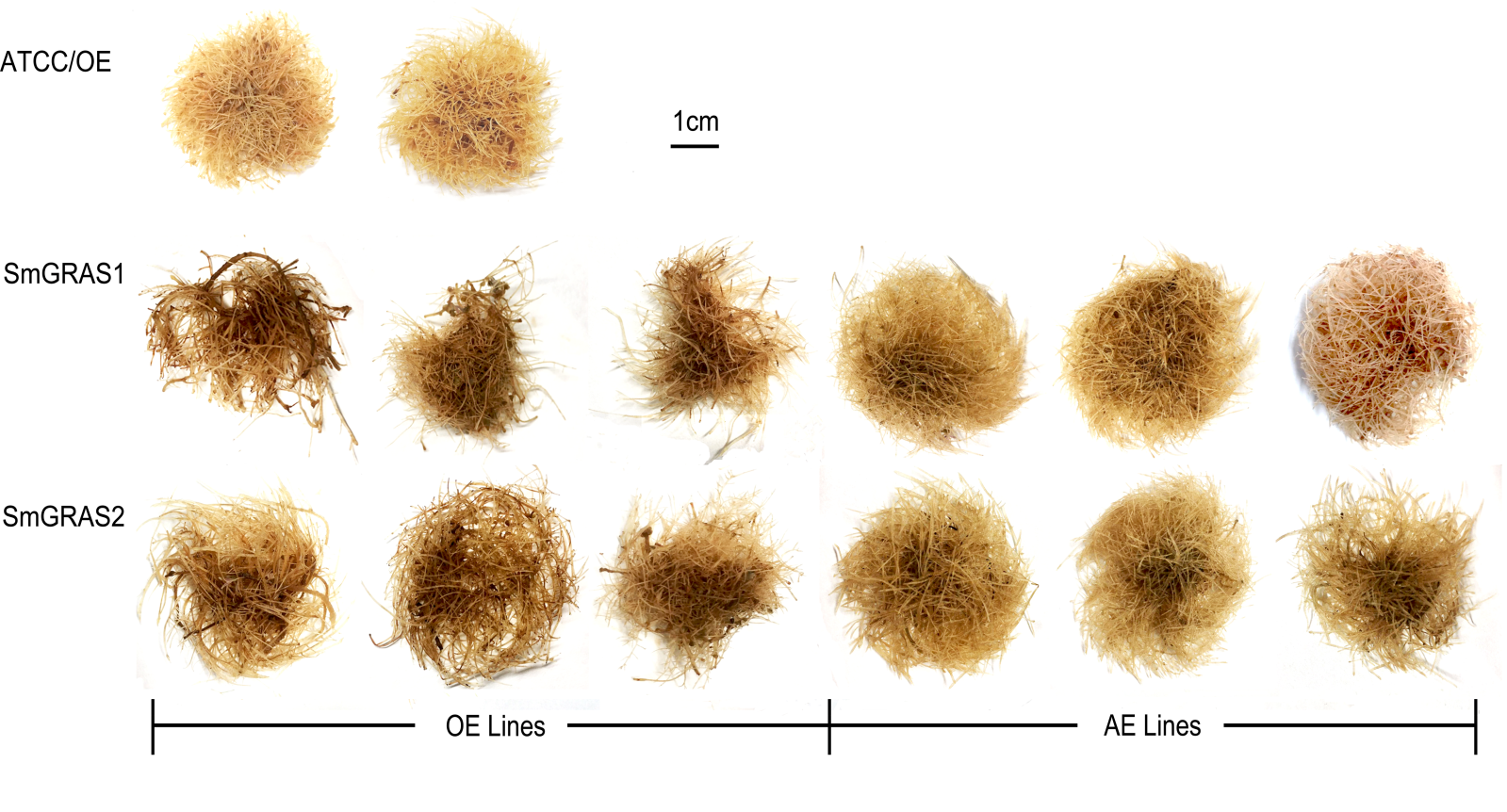


**FIGURE S4 |** Growth phenotypes of *SmGRAS1/2* transgenic hairy roots lines.

**
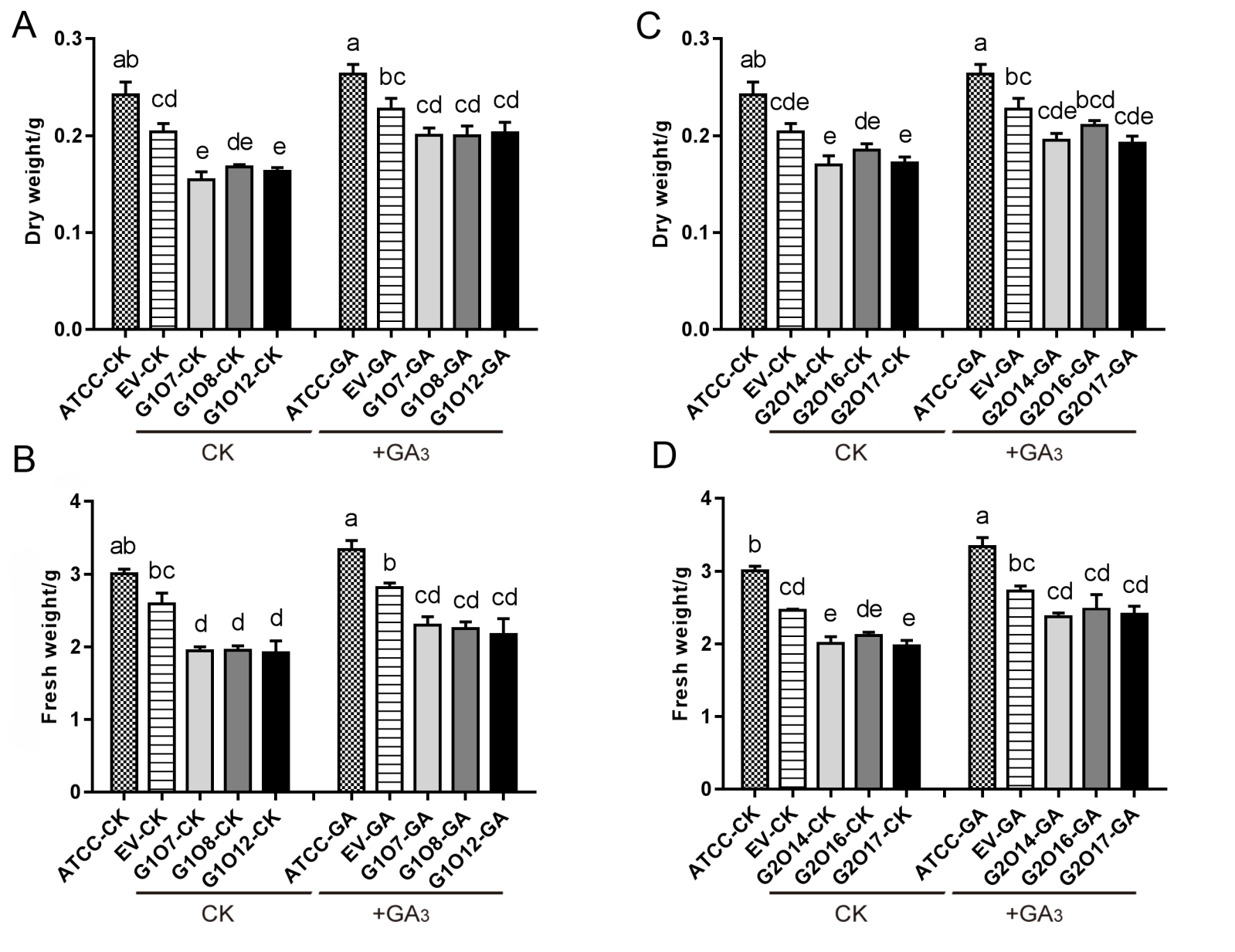
**

**FIGURE S5 |** GA affects the root biomass of *SmGRAS1* and *SmGRAS2* OE lines. **(A,B)** GA affects the root biomass of *SmGRAS1* OE lines. **(C,D)** GA affects the root biomass of *SmGRAS2* OE lines. 21-day-old hairy roots lines treated with 100 μM GA_3_ for 6 days. Standard errors were calculated from three sets of biological replicates. Significant differences using one-way ANOVA and S-N-K comparison tested, *P* < 0.05.

**
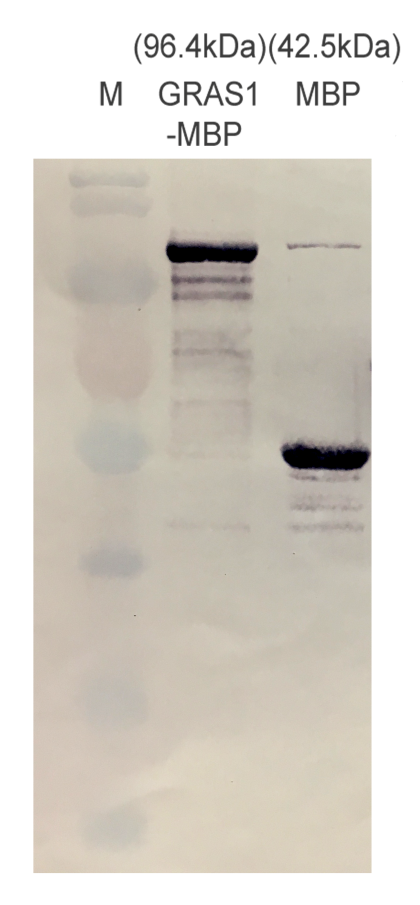
**

**FIGURE S6 |** Purified MBP protein and SmGRAS1 protein with an MBP label were verified via Western blot. M: protein marker.
